# Genomic Features and Pervasive Negative Selection in *Rhodanobacter* Strains Isolated from Nitrate and Heavy Metal Contaminated Aquifer

**DOI:** 10.1128/spectrum.02591-21

**Published:** 2022-02-02

**Authors:** Mu Peng, Dongyu Wang, Lauren M. Lui, Torben Nielsen, Renmao Tian, Megan L. Kempher, Xuanyu Tao, Chongle Pan, Romy Chakraborty, Adam M. Deutschbauer, Michael P. Thorgersen, Michael W. W. Adams, Matthew W. Fields, Terry C. Hazen, Adam P. Arkin, Aifen Zhou, Jizhong Zhou

**Affiliations:** a Institute for Environmental Genomics, Department of Microbiology and Plant Biology, University of Oklahoma, Norman, Oklahoma, United States; b Department of Microbiology and Plant Biology, University of Oklahoma, Norman, Oklahoma, United States; c Environmental Genomics and Systems Biology Division, Lawrence Berkeley National Laboratory, Berkeley, California, United States; d Earth and Environmental Sciences Area, Lawrence Berkeley National Laboratory, Berkeley, California, United States; e Department of Plant and Microbial Biology, University of California, Berkeley, California, United States; f Department of Biochemistry and Molecular Biology, University of Georgia, Athens, Georgia, United States; g Department of Microbiology and Cell Biology, Montana State University, Bozeman, Montana, United States; h Center for Biofilm Engineering, Montana State University, Bozeman, Montana, United States; i Department of Civil and Environmental Engineering, University of Tennessee, Knoxville, Tennessee, United States; j Oak Ridge National Laboratory, Bioscience Division, Oak Ridge, Tennessee, United States; k Department of Bioengineering, University of California, Berkeley, California, United States; l State Key Joint Laboratory of Environment Simulation and Pollution Control, School of Environment, Tsinghua University, Beijing, China; m School of Civil Engineering and Environmental Sciences, University of Oklahoma, Norman, Oklahoma, United States; University of Massachusetts Amherst

**Keywords:** *Rhodanobacter*, comparative genomics, methylation, negative selection, horizontal gene transfer, restriction-modification system genes

## Abstract

*Rhodanobacter* species dominate in the Oak Ridge Reservation (ORR) subsurface environments contaminated with acids, nitrate, metal radionuclides, and other heavy metals. To uncover the genomic features underlying adaptations to these mixed-waste environments and to guide genetic tool development, we sequenced the whole genomes of eight *Rhodanobacter* strains isolated from the ORR site. The genome sizes ranged from 3.9 to 4.2 Mb harboring 3,695 to 4,035 protein-coding genes and GC contents approximately 67%. Seven strains were classified as *R. denitrificans* and one strain, FW510-R12, as *R. thiooxydans* based on full length 16S rRNA sequences. According to gene annotation, the top two Cluster of Orthologous Groups (COGs) with high pan-genome expansion rates (Pan/Core gene ratio) were “replication, recombination and repair” and “defense mechanisms.” The denitrifying genes had high DNA homologies except the predicted protein structure variances in NosZ. In contrast, heavy metal resistance genes were diverse with between 7 to 34% of them were located in genomic islands, and these results suggested origins from horizontal gene transfer. Analysis of the methylation patterns in four strains revealed the unique 5mC methylation motifs. Most orthologs (78%) had ratios of nonsynonymous to synonymous substitutions (dN/dS) less than one when compared to the type strain 2APBS1, suggesting the prevalence of negative selection. Overall, the results provide evidence for the important roles of horizontal gene transfer and negative selection in genomic adaptation at the contaminated field site. The complex restriction-modification system genes and the unique methylation motifs in *Rhodanobacter* strains suggest the potential recalcitrance to genetic manipulation.

**IMPORTANCE** Despite the dominance of *Rhodanobacter* species in the subsurface of the contaminated Oak Ridge Reservation (ORR) site, very little is known about the mechanisms underlying their adaptions to the various stressors present at ORR. Recently, multiple *Rhodanobacter* strains have been isolated from the ORR groundwater samples from several wells with varying geochemical properties. Using Illumina, PacBio, and Oxford Nanopore sequencing platforms, we obtained the whole genome sequences of eight *Rhodanobacter* strains. Comparison of the whole genomes demonstrated the genetic diversity, and analysis of the long nanopore reads revealed the heterogeneity of methylation patterns in strains isolated from the same well. Although all strains contained a complete set of denitrifying genes, the predicted tertiary structures of NosZ differed. The sequence comparison results demonstrate the important roles of horizontal gene transfer and negative selection in adaptation. In addition, these strains may be recalcitrant to genetic manipulation due to the complex restriction-modification systems and methylations.

## INTRODUCTION

*Rhodanobacter* species have been found in diverse habitats such as groundwater (1), sediments (2), soil (3, 4), and as a plant endophyte (5). One of the important features of *Rhodanobacter* species is the capability of complete denitrification (1). In addition, *Rhodanobacter* species have been shown to be metal resistant (6). *Rhodanobacter* species dominance has been observed in the most metal-contaminated aquifers and the acidic source zone areas at the U.S. Department of Energy’s Oak Ridge Reservation (ORR) site (7). The existence of abundant metal resistance genes in *Rhodanobacter* strains has been demonstrated by metagenomic data of the ORR site groundwater samples (8). As most studies have been based on metagenomic sequencing, studies with culture-dependent methods (i.e., isolation and growth) are needed to understand the growth, physiology, and mechanisms of adaptation for discrete but related populations (9).

Comparative genomic analysis, which compares protein-encoding genes and regulatory regions between multiple genomes, can uncover the genetic diversity and reveal the possible mechanisms underlying the genetic differences, adaptability, and evolution (10). For instance, a comparative genomics analysis of *Acidithiobacillus* spp. revealed the evolutionary history of metal resistance genes (11). The comparative genomic analysis of Actinetobacter baumannii and Enterococcus faecium uncovered extensive genomic variation, evolution, and niche adaptation in different hosts or environments (12, 13). As another example, 11 nitrogen-cycling pathways were identified by the comparative genomic analysis of over 6,000 complete bacterial and archaeal genomes (14). So far, seven *Rhodanobacter* species encompassing 76 strains have been sequenced, and the complete genome sequences of three strains are available in the NCBI database (NC_020541.1 for R. denitrificans 2APBS1, NZ_CP042807 for R. glycinis, and NZ_CP069535 for *Rhodanobacter* sp.). Among these strains, 2APBS1 was isolated from ORR. Given the dominance of *Rhodanobacter* species at the ORR site, the comparative genome analysis of multiple *Rhodanobacter* species would provide valuable insights about their adaptation and evolutionary mechanisms in stressful environments at ORR.

By combining short read Illumina sequencing and long read sequencing such as Nanopore or PacBio, we aimed to obtain the whole genome sequences of the *Rhodanobacter* species from ORR and identify the methylation profiles. Methyltransferases (MTase) catalyze the transfer of a methyl group from S-adenosyl-L-methionine (SAM) to the appropriate position on target bases, resulting in three different forms of DNA methylation including N6-methyladenine (6mA), N4-methylcytosine (4mC), and 5-methylcytosine (5mC) in bacterial genomes. The bacterial cells are protected by cleaving nonmethylated foreign DNA but not methylated endogenous DNA via the function of methyltransferase and restriction enzymes, namely, the restriction-modification systems. The MTases specificity domains and the target motifs vary across species, resulting in high diversity of methylation spectrums. However, the precise sequence motifs of methylation in most microorganisms remain unknown. DNA methylation is a primary mechanism of epigenetic gene regulation in bacteria (15), and heterogeneity in methylation patterns within bacterial populations promotes adaptive selection by resulting in heterogeneity in gene expression and cellular phenotypes (16). The recently developed sequencing technologies, such as nanopore sequencing, and the related machine learning tools, enable the identification of DNA methylations based on the electrical signal changes due to the epigenetic changes in the nucleotides (15, 17). Identification of the prevalent methylations 5mC and 6 mA and the conserved methylation motifs of *Rhodanobacter* strains could provide valuable information related to the adaptation mechanisms.

Taking advantage of the availability of eight *Rhodanobacter* strains isolated from the ORR site, we conducted whole genome sequencing and comparative genomic analysis. Since all strains were isolated from the ORR site containing large amounts of acids, nitrate, metal radionuclides, and other heavy metals, we aimed to uncover the genetic diversity and genomic structure variations to provide insights into genetic adaptations under environmental stresses and to help guide the future development of genetic manipulation tools for these indigenous strains. The complete set of denitrifying genes, diverse heavy metal resistance genes, abundant genomic islands, and complex restriction-modification system genes were identified in all strains. The high diversity of heavy metal resistance genes and the location of approximately 7% to 34% of heavy metal resistance genes in genomic islands provided genomic-level evidence for the importance of horizontal gene transfer in the adaptation of *Rhodanobacter* species. The heterogeneity of methylation patterns in four strains suggested the important role of methylation in adaptation, and sequence comparison of orthologs in these strains indicated the prevalence of negative selection.

## RESULTS

### General genome features.

Eight *Rhodanobacter* strains were isolated from four different wells at the ORR with different geochemical properties (Table S1 in the supplemental material). The general genomic features and the sequencing platforms are listed in Table S2. The genome sizes ranged from 3.9 Mb to 4.2 Mb, and the numbers of protein-coding genes varied from 3,695 to 4,035. The GC contents were very similar, ranging from 67.36% to 67.66%. The number of proteins with functional assignments, Enzyme Commission (EC) number assignments, Gene Ontology (GO) assignments, or pathway assignments were also very similar among the strains. Three strains including FW104-R5, FW104-MT042, and FW510-R12 had one plasmid. Comparison of the genome of FW107-2APBS1 with the genome sequence of 2APBS1 in the NCBI database demonstrated 18 single nucleotide polymorphisms (SNPs) or small insertion/deletion(s) (1∼2 bp), two insertion/deletions of >400bp, and one big deletion of 0.24 Mb (Table S3). The sequence difference was probably due to the assembling methodology. FW107-2APBS1 genome sequence was used as the reference genome in this study.

### Phylogenetic relationships and overall genome similarities.

**FIG 1:**
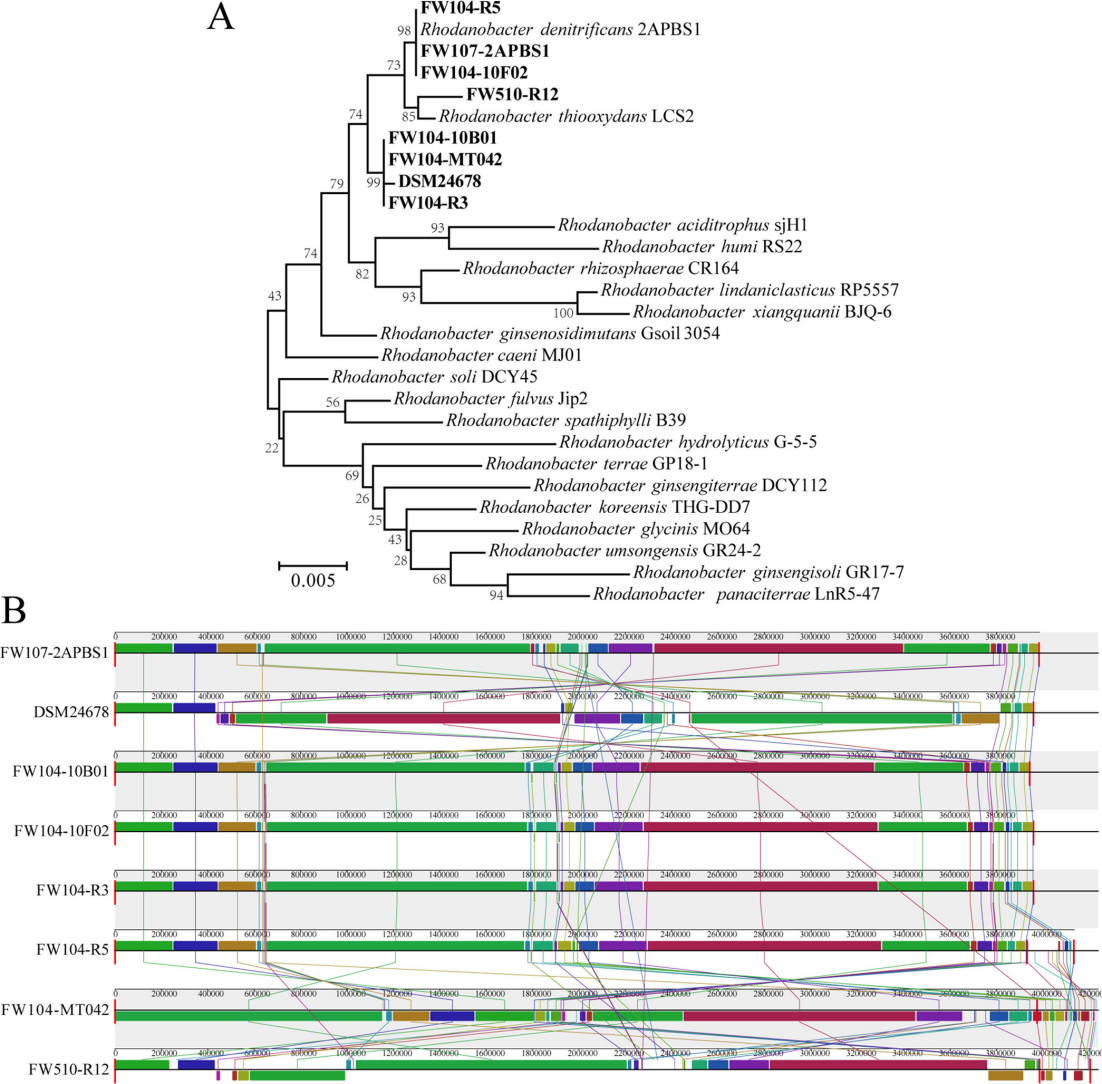
Phylogenetic relationship of eight *Rhodanobacter* strains. (A) Neighbor-joining phylogenetic tree based on 16S rRNA gene sequences using MEGA 6.0. (B) Genome alignment based on MAUVE analysis. The locally colinear blocks (LCBs) represent highly homologous regions and are shown with different colors.

A phylogenetic tree based on full-length 16S rRNA gene sequences or other conserved functional genes is often used for phylogenetic relationship analysis. However, misidentification and misclassification of strains based on 16S rRNA genes have been reported, possibly due to horizontal gene transfer (HGT), gene homology, or the differences of gene evolution rates among species (18–20). Therefore, average amino acid identity (AAI) and average nucleotide identity (ANI) of orthologous genes are often used to identify the phylogenetic relationships. An AAI > 96% and ANI > 95% are considered to be the same species (21, 22). Here we analyzed the phylogenetic relationships of these *Rhodanobacter* strains using 16S rRNA gene similarity, AAI, and ANI. In the 16S rRNA gene-based phylogenetic tree (Fig. 1A), four strains including DSM24678, FW104-R3, FW104-MT042, and FW104-10B01 clustered together as one clade. FW107-2APBS1, FW104-10F02, FW104-R5 clustered together as one clade, and strain FW510-R12 clustered with R. thiooxydans LCS2 in another clade. The ANI and AAI values indicated that seven strains belong to the same species; strain FW510-R12 was the exception (Table S4), with the AAI and ANI values lower than 90%, consistent with the 16S rRNA gene analysis (Fig. 1A). In addition, very high degrees of similarities of both ANI and AAI (>99%) were found in four out of five strains isolated from well FW104 except for FW104-MT042.

At the whole genome level, genome alignment is a powerful tool to identify the conserved or unique regions and provide genome-level evidence for biological behavior or adaptations to specific ecological niches (23). Genome alignment with MAUVE showed that most of the locally colinear blocks (LCBs) were highly homologous, suggesting that there is an overall colinear relationship among strains (Fig. 1B). The chromosomal arrangements of some LCBs were quite different, and multiple inversions were observed. R. thiooxydans FW510-R12 shows very low collinearity and more genomic rearrangements than the other strains. The synteny plot, usually used to show the conservation of gene order among genomes and large-scale genome rearrangements, also showed that all genomes exhibit highly conserved gene order except strain FW510-R12 (Fig. S1). Taken together, the results demonstrate the overall similarities among seven R. denitrificans strains and the differences between R. denitrificans strains and the R. thiooxydans strain FW510-R12.

### Pan-genome and core genome.

**FIG 2:**
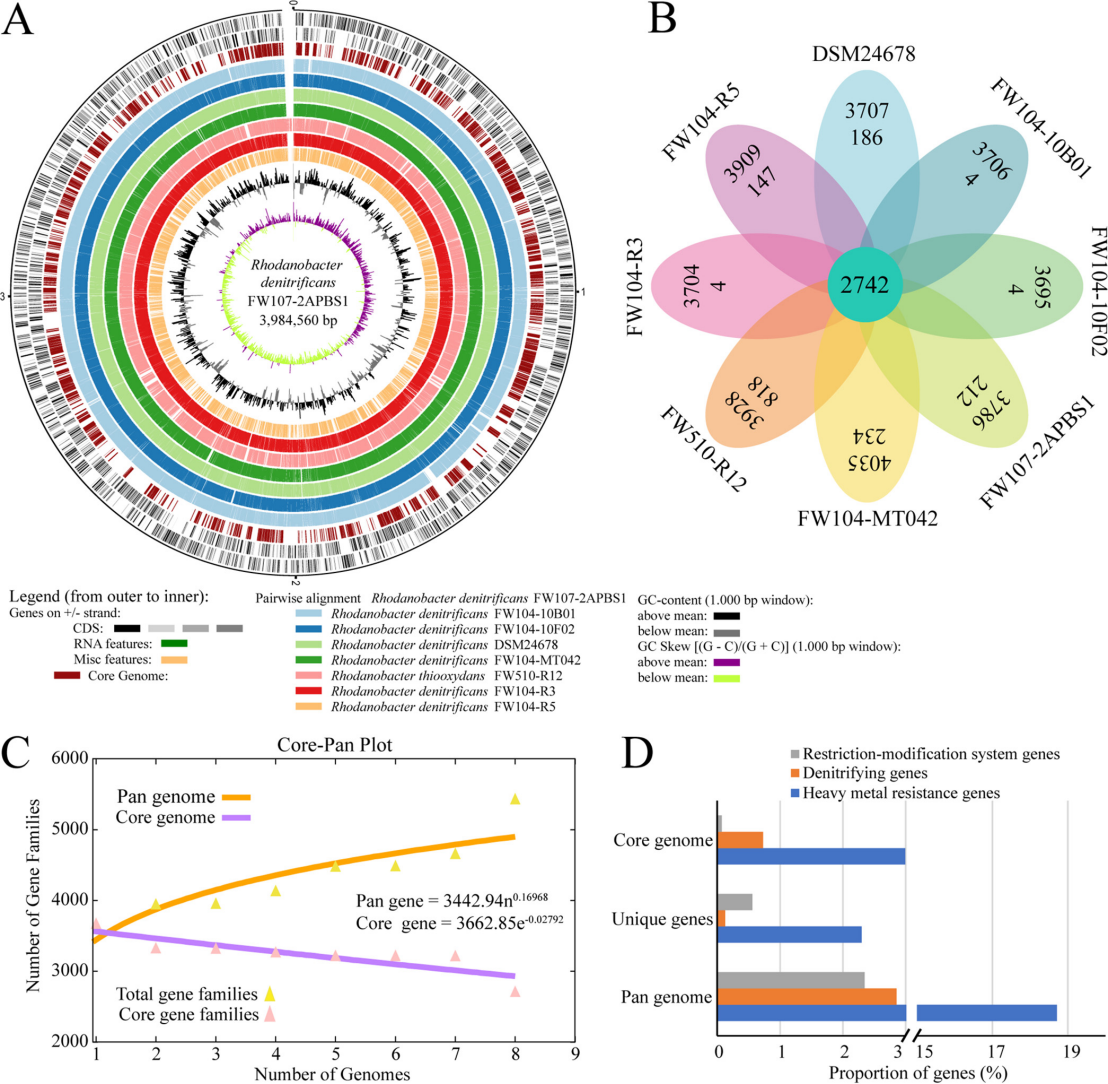
Comparative genomic analysis of *Rhodanobacter* strains. (A) Circular genome comparison showing the CDS, core genome, pairwise alignment, and GC content. (B) Venn diagram of the pan-genome. The numbers in the outer ring, inner ring, and center represent the numbers of protein-coding genes, the unique genes, and the core genes, respectively. (C) Accumulation curves of the pan- and core-genomes. (D) Proportions of genes involved in denitrification processes, heavy metal resistance, and restriction-modification system in pan genome, core genome, or unique genes.

To further investigate the similarities among the genomes, the annotated genes were analyzed using pairwise alignment. Most of the genes were conserved without large fragment deletions or insertions (Fig. 2A). A total of 2,742 genes were identified as core genome, and 1,609 genes were unique genes (Fig. 2B). Over 69.8% of the protein-coding genes in each strain were core genes, which was higher compared to the less than 50% core genes in most bacteria at different taxonomic levels (24). As the core genome size calculation is related to parameters such as the number of genomes, selection of strains, the orthologous cut-off values, and various classification levels (25), we interpreted that the relative higher portion of core genes in these *Rhodanobacter* strains is probably due to the small number of genomes included in this study or the high similarity among these genomes from a single field site with strong selection (i.e., nitrates, metals, low pH). In terms of the unique genes, *R. thiooxydans* strain FW510-R12 had the greatest number (818) of unique genes. Among the seven *R. denitrificans* strains, the numbers of unique genes in strains from well FW104 varied with the least number of unique genes (4) in strains FW104-10B01, FW104-10F02, and FW104-R3; the intermediate number of unique genes (147) in FW104-R5; and the highest number of unique genes (234) in FW104-MT042 (Fig. 2B). The varying number of unique genes in these genomes may be an outcome of survival in the extreme environment where microniches might exist and could contribute to the large pan-genome pools, as bacterial genomes are constantly evolving via gene gain, gene loss, and genomic rearrangements (26).

At the gene function level based on Cluster of Orthologous Groups (COG), the pan-genome and core-genome have different gene distribution patterns. For COGs with annotated specific functions, the top three COGs in the core genome were “amino acid transport and metabolism,” “energy production and conversion,” and “translation, ribosomal structure and biogenesis” (Fig. S2). While the top three COGs in the pan-genome were “transcription,” “replication, recombination and repair,” and “cell wall/membrane/envelope biogenesis.” Many COGs had high pan-genome expansion rates (the ratio of Pan/Core genes, Fig. S2). For instance, the highest pan-genome expansion rate of 4.5 was observed in COG of “replication, recombination and repair.” Five out of seven COGs in “cellular processes and signaling pathways” and six out of eight COGs in “metabolism” had pan-genome expansion rates greater than 1.2. Among these expanded COGs, expansion of pan genes in “defense mechanisms” (expansion rate 4.3) and “inorganic ion transport and metabolism” (expansion rate 2.6) might be the consequences of adaptation of *Rhodanobacter* species to the stressors such as nitrate, acid, and heavy metals at the ORR site, as “defense mechanisms” genes are accessory genes and likely associated with adaptations to environmental stressors (27, 28). The large extents of pan-genome expansion indicated the roles of these genes in adaption to the stressful environment.

The genome accumulation curves indicated that the pan-genome size increased continuously (γ = 0.170), while the core genome size gradually stabilized (−n/τc = −0.028) as the number of genomes increased (Fig. 2C). The power law regression function for pan gene was *Ps = *3442.94*n*^0.16968^, where *γ* = 0.16968. γ > 0 indicates that these *Rhodanobacter* species have an open pan-genome, and a considerable number of unique genes are expected to be detected as more genomes are sequenced. Bacterial species with an open pan-genome can colonize and exploit a wide range of environments and expand accessory functions and pan-genome by horizontal gene transfer (29). The open pan-genome of *Rhodanobacter* species demonstrated their genetic diversity and partly explained the selective advantages in a multistressor environment.

Considering the capability of complete denitrification of most *Rhodanobacter* species (1) and the presence of multiple stressors in the sampling wells such as high concentrations of heavy metals (Table S1), a survey of genes involved in these processes demonstrated that the pan genome had a relatively higher portion of genes involved in denitrification, heavy metal resistance, and restriction-modification system genes than the core genome and unique genes (Fig. 2D). Comparison of these genes across these genomes as well as methylation patterns are described below.

### Denitrifying genes.

**FIG 3:**
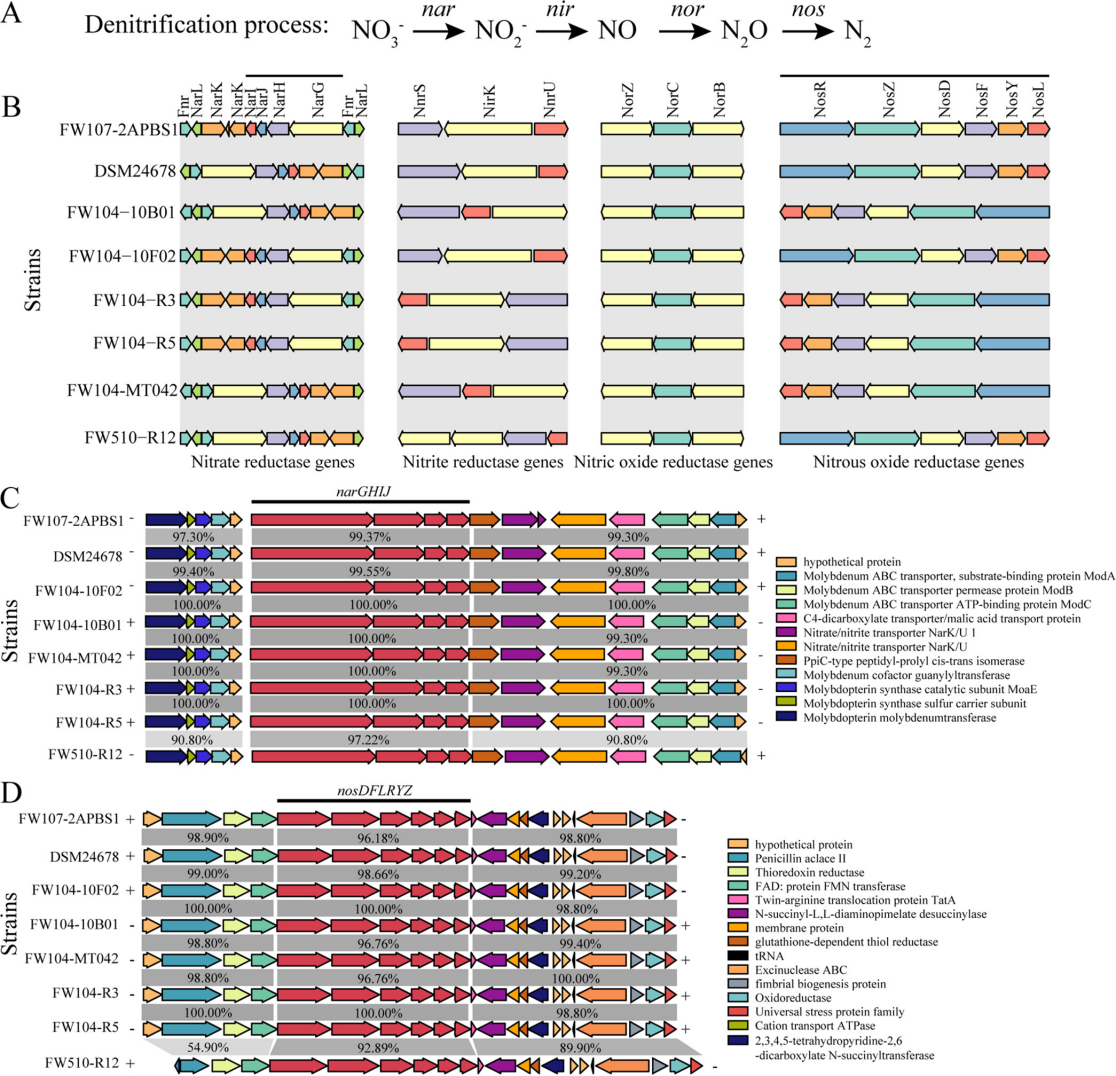
Chromosomal distribution and organization of the genes involved in denitrification processes. (A) The schematic representation of the denitrification process and the associated genes. (B) The chromosomal organizations of genes involved in four steps of denitrification. (C and D) Synteny of the regions flanking the nitrate reductase gene (*narGHIJ*) and nitrous reductase gene (*nosDFLRYZ*) clusters, respectively. The percentages indicate the amino acid identity with FW107-2APBS1 as reference. Arrows indicate the direction of transcription. Homologous genes are highlighted by the same color. Black lines represent gene clusters.

The ORR groundwater has some of the highest nitrate levels reported in the world, and in some cases it can be 10s of grams per liter (10,000× above U.S. EPA drinking water levels). The genes involved in the four steps of denitrification, namely, nitrate reductase (*nar*), nitrite reductase (*nir*), nitric-oxide reductase (*nor*), and nitrous oxide reductase (*nos*) (Fig. 3A), were identified in all genomes except that the chromosomal distribution and organization of these genes varied in the different genomes (Fig. 3B). In addition to the membrane-bound nitrate reductases (Nar), periplasmic nitrate reductase (Nap) has been reported to catalyze the reduction of nitrate to nitrite in bacteria (30). In these *Rhodanobacter* genomes, no *nap* genes were identified. All genomes had four *nar* genes; three *nir* genes except that there was an extra copy of *nirK* in the FW510-R12 genome; three *nor* genes; and six *nos* genes. Among these genes, only *narGHIJ* and *nosDFLRYZ* genes formed clusters; all other genes were in different genome loci.

**FIG 4:**
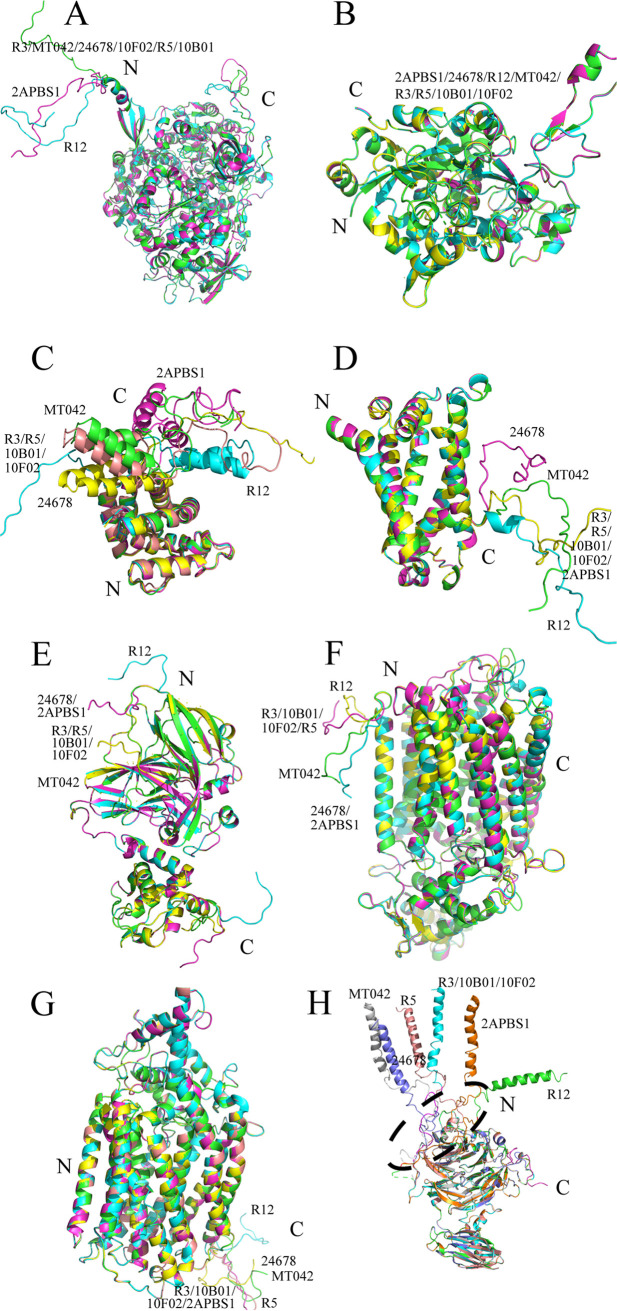
Comparison of the tertiary structures of the key proteins involved in the denitrification process. Protein structures were predicted by RaptorX (raptorx.uchicago.edu). (A) NarG. (B) NarH. (C) NarI. (D) NarK. (E) NirK. (F) NorZ. (G) NorB. (H) NosZ. Strain names: 24678: DSM24678; 10F02: FW104-10F02; R3: FW104-R3; R5: FW104-R5; 10B01: FW104-10B01; R12: FW510-R12; MT042: FW104-MT042; 2APBS1: FW107-2APBS1.

To gain insight into the similarities of *nar* and *nos* gene clusters among the eight genomes, the DNA sequences of the gene clusters and the flanking regions were compared (Fig. 3C and D). The coding sequence identities of both gene clusters were over 96% in all strains except FW510-R12. The flanking regions of both gene clusters were also highly conserved (>89.9% similarity) except the low similarity (54.9%) in upstream region of *nosDFLRYZ* in FW510-R12. At the protein level (Fig. 4), the predicted protein structures of the key proteins in the denitrification steps were similar and highly overlapped, except the N-terminal coils in NosZ, suggesting that the function or activity of NosZ in FW510-R12 may differ from that in the seven *R. denitrificans* strains.

### Heavy metal resistance genes and genomic islands.

**FIG 5:**
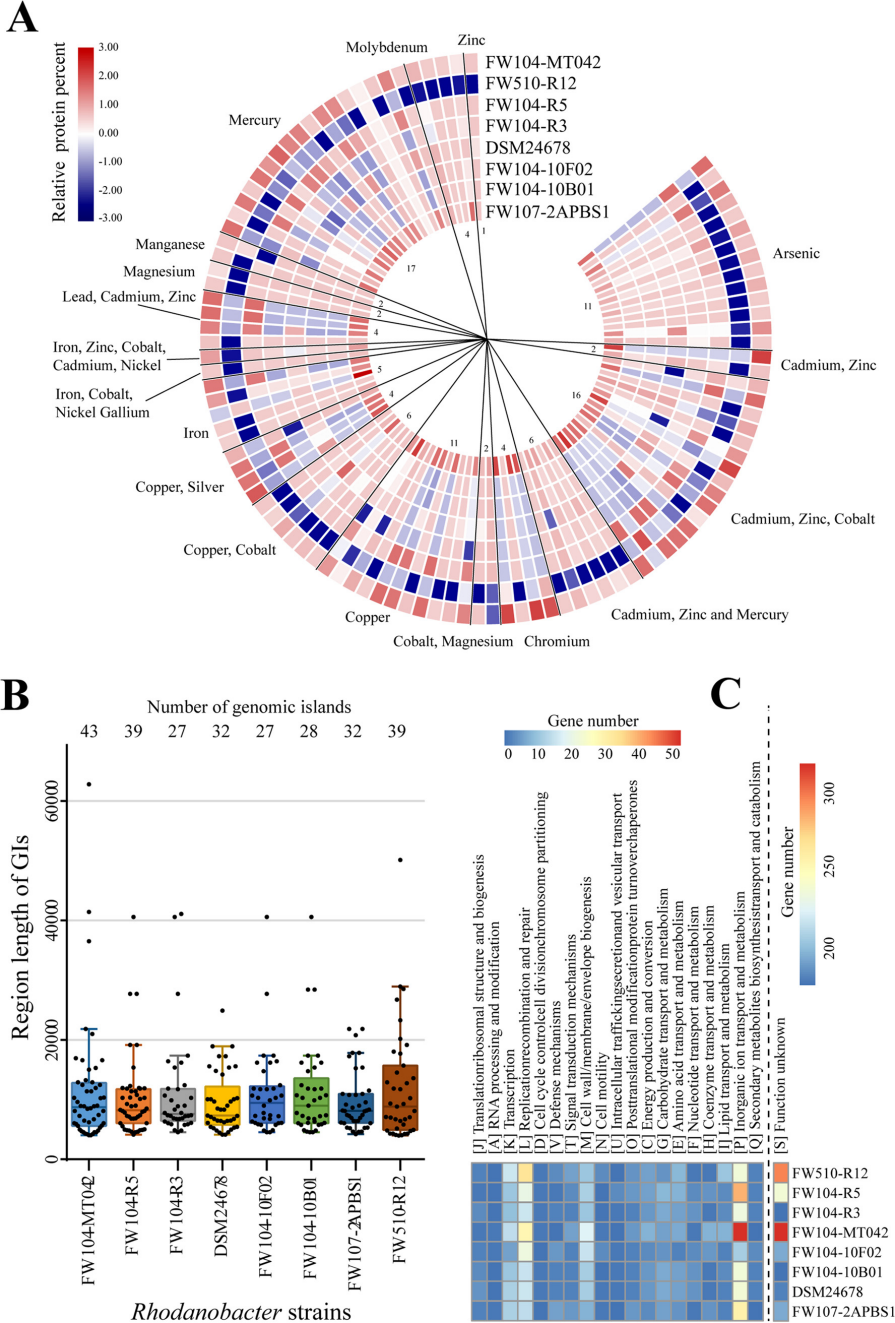
Protein sequence comparison of the metal resistance genes (A) and summary of genomic islands (GIs) (B and C) in eight *Rhodanobacter* genomes. (A) Each circle represents one strain and each box shows one protein. The color of the box represents the amino acid sequence identity with FW107-2APBS1 as the reference. (B) The numbers and lengths of GIs. (C) The functional annotation of genes located in GIs based on EggNOG database.

An average of 135 heavy metal resistance genes belonging to 18 groups were identified in each genome (Fig. 5A and Table S5). Strain FW510-R12 had the lowest number (125) of metal resistance genes and the lowest similarities to genes in other strains. Interestingly, metal resistance genes encoding predicted cobalt/zinc/cadmium resistance proteins, arsenate reductase, arsenical resistance proteins, mercuric transport proteins, and high-affinity iron permease were located in genomic islands (GIs) (Table S5), suggesting their possible origins of horizontal gene transfer (HGT), which can play a vital role in genome evolution (31, 32). The numbers of GIs in each genome ranged from 27 to 43, and the lengths of GIs varied from 4,004 bp to 62,764 bp (Fig. 5B). Most genes (1,705 out of 2,845; Table S5) in GI encode hypothetical proteins. Among genes with annotated specific function, the greatest number of genes were in COG “inorganic ion transport and metabolism” where metal resistance genes are typically classified (Fig. 5C). The numbers of GI-located metal resistance genes in each strain varied, with 29 in 2APBS1, 51 in FW104-MT042, 30 in FW104-R5, 11 in FW104-R3, 8 in DSM24678, 11 in FW104-10F02, 11 in FW104-10B01, and 18 in FW510-R12 (Table S5), corresponding to 21%, 34%, 22%, 8%, 7%, 8%, 8%, and 14% of the total numbers of metal resistance genes, respectively. The varied numbers of GI-located metal resistance genes suggested that the origins of these genes were probably from different and distantly related species via HGT.

### Restriction-modification system genes.

**FIG 6:**
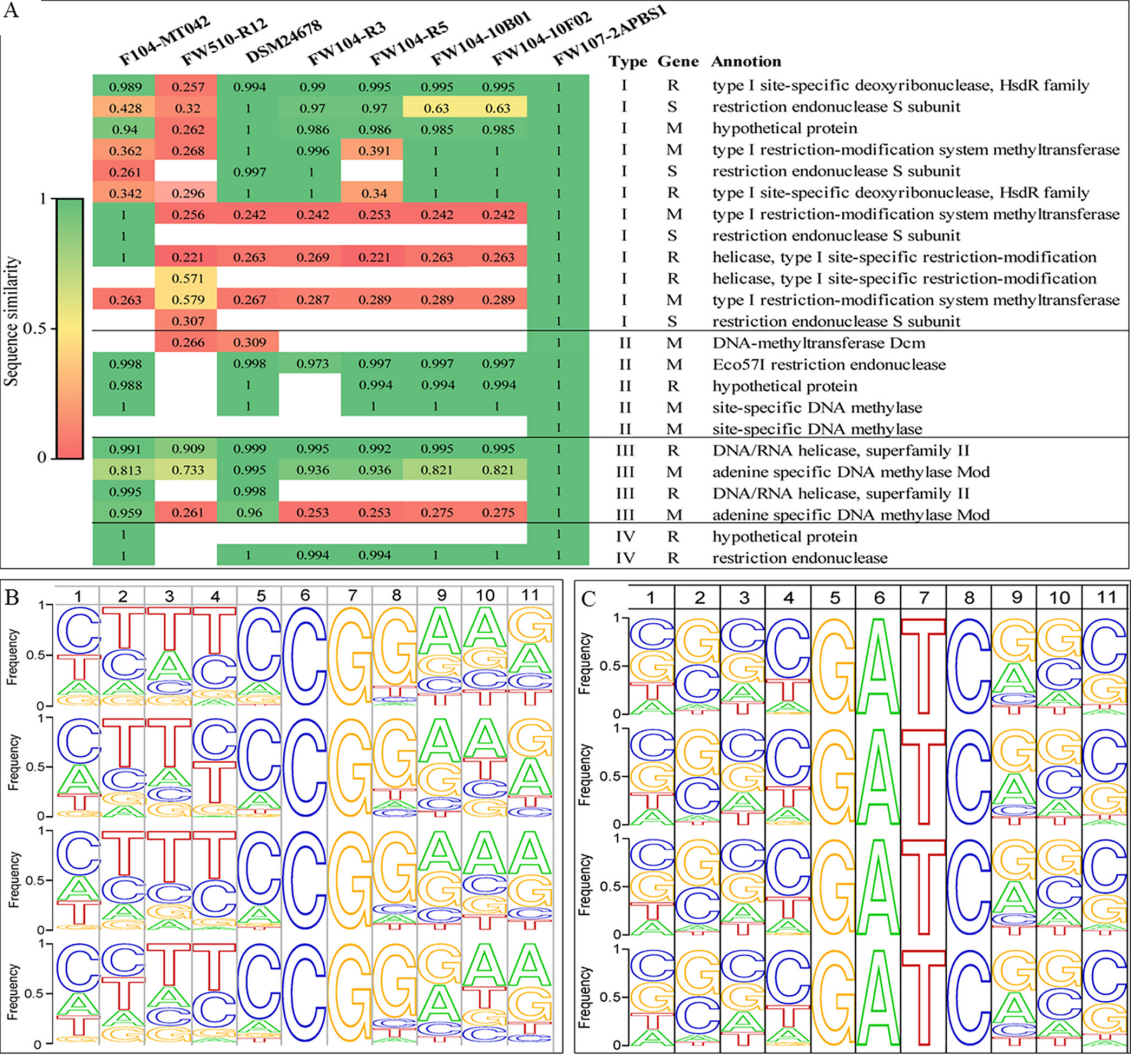
Restriction-modification system genes (A) and methylation motifs of 5mC (B) and 6mA (C) in four *Rhodanobacter* genomes. FW107-2APBS1 was used as the reference for the percent amino acid identity of the restriction-modification system genes (A). The methylation motifs in four *R. denitrificans* genomes (from the top to the bottom: DSM24678, FW104-10F02, FW104-R3, and FW104-R5).

Restriction-modification (RM) systems are host defense systems in microorganisms against exogenous DNA via recognizing and cleaving unmethylated foreign DNA while the methylated host DNA is protected from cleavage (33). RM systems are classified into types I to type IV based on enzyme subunit composition, cofactor requirements, and DNA specificity and reaction products (34). Type I to IV system RM genes were annotated in all genomes, and about half of them belonged to type I RM genes (Fig. 6A). Strain FW107-2APBS1 had the greatest number (23), and FW510-R12 had the least number (14) of RM genes. With FW107-2APBS1 as the reference, the RM genes were highly conserved among all strains except FW510-R12. The RM systems facilitate adaptive evolution by promoting DNA recombination and contributing to genetic variation in microbial populations (35, 36) and are also considered as barriers against genetic transformation (37). Type I RM systems are complex and notable for the ability to evolve new species (38). Therefore, the existence of abundant and diverse RM system genes in these *Rhodanobacter* strains might result from population selection in a multistressor environment and potentially lead to recalcitrance of genetic manipulation.

### Methylation patterns.

DNA methylation profiles including 5-methylcytosine (5mC) and N6-methyladenine (6mA) were identified by analyzing the nanopore sequences of four genomes including three strains from FW104 and one strain (DSM24678) from the most severely contaminated area. The total genome lengths of all genomes were covered (100%) by the nanopore reads (Table S6). When aligned to the assemblies of the Illumina sequences, the average identity and the read accuracy per aligned nanopore read were above 0.96 and 97% respectively (Fig. S3), both of which were comparable to or higher than the values reported in literature (39), indicating the high quality of the nanopore reads. The numbers of 5mC methylation sites ranged from 52 to 63 in the genomes, and the locations of most 5mC (71%∼81%) were in open reading frames (ORF), about 14%∼29% in promoter regions (<150 bp upstream of start codon), and very few in intergeneric regions (Table 1, Table S7). Alignment of the 5mC methylation sites indicated the conserved dinucleotide CG in the center with less conserved flanking bases (Fig. 6B). The methylation sites represented about 0.025% to 0.046% of the total CG dinucleotides or about 0.004% of C in the genomes. In contrast, about 900 to 1,100 6 mA were identified (Table 1, Table S8) with locations mostly in intergenic regions. Alignment of the 6mA motifs indicated the conserved motif of G^m^ATC in the center and the less conserved flanking bases (Fig. 6C). The methylation sites represented 0.83%∼1.66% of the total tetra-nucleotide GATC or 0.14%∼0.18% of A in the genomes. In addition to the sequence analysis, restriction enzyme digestion of genomic DNA or plasmid DNA with DpnI or DpnII was conducted. DpnI cleaves methylated adenine to thymine in the 5′ to 3′ direction in motif G^m^A|TC, while DpnII cleaves ^▾^GATC_▴_ without methylation. Genomic DNA from five *Rhodanobacter* strains and plasmid DNA isolated from strain FW107-2APBS1 were digested by DpnII but not DpnI; in contrast, the plasmid DNA isolated from E. coli was digested by DpnI but not DpnII (Fig. S4). Considering the existence of G^m^ATC and CpG in the genomes, the enzyme digestion results suggested the possible overlapping of G^m^ATC and CpG in the genomes. Adenine specific DNA methylases *mod* were annotated in these genomes (Fig. 6) but methyltransferase gene *dcm* for 5mC methylation was only annotated in strain DSM24678, indicating the complexity of the restriction-modification systems in *Rhodanobacter* genomes.

**TABLE 1:** Genome locations of methylated C or A and the abundances of 5mC or 6 mA in the whole genome

Genome	Promoter	%	ORF	%	Intergeneric	%	# of mCG	# of CG in genome	% of mCG	# of C in genome	% of 5mC
5mC											
DSM24678	15	28.85%	37	71.15%			52	206,654	0.025%	1,345,709	0.0039%
FW104-10F02	10	16.95%	48	81.36%	1	1.69%	59	129,110	0.046%	1,341,059	0.0044%
FW104-R3	8	14.04%	46	80.70%	3	5.26%	57	159,926	0.036%	1,337,342	0.0043%
FW104-R5	13	20.63%	50	79.37%			63	155,175	0.041%	1,395,819	0.0045%
6mA											
DSM24678	44	4.88%	214	23.75%	643	71.37%	901	108,725	0.83%	642,657	0.14%
FW104-10F02	53	4.67%	290	25.55%	792	69.78%	1,135	68,562	1.66%	638,224	0.18%
FW104-R3	49	4.65%	294	27.89%	711	67.46%	1,054	83,234	1.27%	642,486	0.16%
FW104-R5	27	2.66%	493	48.52%	496	48.82%	1,016	81,339	1.25%	670,204	0.15%

### Negative selection.

To identify whether positive selection, negative selection, or neutral selection played a major role in the evolutionary adaptation of these *Rhodanobacter* strains, pairwise analysis of the ratios of nonsynonymous to synonymous substitutions (dN/dS) was conducted with FW107-2ABPS1 as a reference. Almost all orthologous genes had dN/dS ratios less than 1 (Table 2), indicating pervasive negative selection. The dN/dS values for all denitrifying genes, metal resistance genes, and one RM gene were less than 1 (Table S9). A strong purifying selective pressure may be beneficial for maintaining the advantageous features in these genomes, particularly in the presence of multistressors and the associated fitness costs. The results indicate that negative selection was one of the main forces driving the evolution of these *Rhodanobacter* species.

**TABLE 2:** Summary of the non-synonymous substitution and synonymous substitutions in orthologous genes with FW107-2APBS1 as reference

Genome	# of orthologs	Without substitutions	Positive selection (dN/dS > 1)	Negative selection (dN/dS < 1)	Neutral selection (dN/dS = 1)
2APBS1-10B01	3,385	613	202	2,570	0
2APBS1-24678	3,380	611	212	2,557	0
2APBS1-MT042	3,447	1011	186	2,250	0
2APBS1-R3	3,381	613	200	2,568	0
2APBS1-R5	3,406	624	203	2,579	0
2APBS1-R12	2,807	11	8	2,788	0
2APBS1-10F02	3,382	612	202	2,568	0
Core genes	2,742	22	13	2,729	0

## DISCUSSION

Combining short reads from Illumina sequencing and long reads from MinION nanopore or PacBio sequencing, whole genomes of eight *Rhodanobacter* strains were obtained. Seven strains were identified as *R. denitrificans*, and one strain FW510-R12 was *R. thiooxydans*. Analysis of the genomes indicated that they have an open pan-genome. All strains had a complete set of denitrifying genes, although the function of *nosZ* might differ in FW510-R12. Genomics features related to adaptative evolution such as diverse heavy metal resistance genes, abundant genomic islands, and complex restriction-modification system genes were identified. 5mC and 6mA profiles and the distinct 5mC methylation motifs were identified in four strains, suggesting the importance of methylation in addition to HGT and negative selection in genomic adaption of *Rhodanobacter* species in a highly contaminated, multistressor environment.

Selection, gene duplication, and horizontal gene transfer (HGT) have been considered the main mechanisms driving genomic adaptation to a changing environment (40, 41). Although both positive selection and negative selection are pervasive in modern evolutionary genetics, negative selection has been proposed as a null model for explaining the genetic diversity (42). Here we observed that the ratios of nonsynonymous to synonymous substitutions (dN/dS) of almost all orthologous genes were less than one, demonstrating that negative selection is pervasive in these *Rhodanobacter* strains, consistent with the findings with metagenomic sequences of the groundwater samples from ORR (43) which explained the decreased diversity in contaminated groundwater compared to pristine groundwater samples (8). In addition to negative selection, the important role of HGT in genomic adaptation of these *Rhodanobacter* species was supported by several lines of evidence. First, there were varying numbers of unique genes in the respective genomes (Fig. 2), and an increased number of unique genes has been suggested as evidence of microbial HGT (44). Second, there are diverse heavy metal resistance genes in each genome, and approximately 7% to 34% of them are localized in GIs. It has been reported that many genes involved in geochemical resistance were horizontally transferred within the community in response to extreme environmental conditions (8, 11).

DNA methylation in bacterial genomes is important for defense against foreign DNA, regulation of gene expression, and population evolution (45, 46). However, knowledge about methylation patterns, the conserved methylation motifs, and the associated DNA methyltransferases is very limited (47). The best-known examples are Dam (DNA adenine MTase) and Dcm (DNA cytosine MTase), which methylate 5′-GATC-3′ and CCWGG motifs, respectively. Based on genome annotations of four strains analyzed in this study, DSM24678 harbored *dcm* and all strains had adenine specific DNA methylase Mod gene (Fig. 6). The conserved motifs were CG for 5mC and GATC for 6mA respectively, both of which had less conserved flanking bases (Fig. 6). The 5mC motif was similar to the methylation motif 5′-CmCGG-3′ of NgoAXIV in Neisseria gonorrhoeae (48). The 6mA methylation motifs were different from that of Mod methyltransferases in Neisseria meningitidis (49). In terms of methylation frequency, bacterial genomes generally have higher levels of 6mA and lower levels of 5mC; frequencies range from undetectable to ∼3% for 6mA and from undetectable to ∼2% for 5mC (50). The frequencies for 6mA and 5mC in four *R. denitrificans* were 0.14%-0.18% and ∼0.004%, respectively, which were much lower than 1.7%∼2.4% for 6mA and 0.92%-0.95% for 5mC in E. coli (50). The locations of the majority of 6mA locations in intergeneric regions and promoter regions suggested the potential roles of methylation in gene expression regulation. Further comprehensive studies of the methylomes of *Rhodanobacter* strains at different growth stages or conditions will reveal the relationships between DNA methylation and the associated RM genes in bacterial genomic adaptation under stressful environment.

Taken together, comparisons among eight *Rhodanobacter* genomes demonstrated the importance of negative selection and HGT underlying the genomic adaptation in a multistressor environment. Ongoing work at the ORR has demonstrated that *Rhodanobacter* species predominate in groundwater and sediments that are high nitrate, low pH, and high heavy metals, and the representative strains had genomic content that could represent presumptive biochemical capacity for denitrification and heavy metal tolerance under low pH. In addition, the complex restriction-modification system genes and the distinct methylation patterns suggest these systems could contribute to fitness and survival in these environments but may lead to recalcitrance to genetic manipulation. Among seven *R. denitrificans* strains, FW104-R3 and DSM24678 had relatively less numbers of RM genes and methylation sites and may be good candidates for genetic tool development. Future work on characterizing the link between individual methyltransferases and the methylome will help remove barriers to the development of genetic editing tools.

## MATERIALS AND METHODS

### Bacterial strains and growth conditions.

Eight *Rhodanobacter* strains isolated from four wells (Table S1) at the U.S. Department of Energy Field Research Center in Oak Ridge (TN, USA) were used for whole genome sequencing. All strains were grown for 2 days at 30°C in R2A medium (Teknova, cat # R0005) and harvested at late exponential phase for genomic DNA isolation.

### Whole genome sequencing, genome assembling, and annotation.

Genomic DNA was extracted using the GenElute Bacterial Genomic DNA Kits (Sigma, Cat # NA2110). Genome sequencing was conducted on an Illumina HiSeq platform (Illumina Inc, USA), a MinION nanopore sequencer (Oxford Nanopore Technologies, United Kingdom), or PacBio. Briefly, for Illumina sequencing library construction, 1 μg of genomic DNA per sample was sheared to 300 bp using a Covaris M220 focused-ultrasonicator (Covaris Inc, MS, USA) before generating the sequencing libraries using KAPA Hyper Prep Kit (KR0961-v2.15, Kapabiosystems). Equal amounts of the DNA library per sample were combined for size selection (300 bp). Finally, the library samples were pooled and loaded for Illumina sequencing. For MinION sequencing library construction, DNA shearing, end-repair, dA-tailing, and adapter ligation procedures were performed with Ligation Sequencing Kit (Oxford Nanopore Technologies, United Kingdom), Covaris microtube (Covaris, USA), NEBNext Ultra II End Repair/dA-Tailing Module (New England Biolabs, USA [NEB]), Blunt/TA Ligase Master Mix (NEB), and Library Loading Bead Kit (Oxford Nanopore Technologies, United Kingdom) according to the manufacturer’s instructions. PacBio sequencing libraries were constructed using SMRT bell Template Prep Kit 1.0 (Pacific Biosciences) and sequenced according to the manufacturer’s instructions.

*De novo* assembly of genomes was performed using a combination of Illumina, nanopore, and PacBio sequencing. Illumina reads were cleaned and trimmed using BBtools (https://jgi.doe.gov/data-and-tools/bbtools) with default parameters. The resulting clean and trimmed Illumina reads were then assembled as a hybrid assembly with either nanopore reads using Unicycler (51) with default parameters or PacBio reads assembled using Flye with default parameters (52) (FW510-R12 and FW104-MT042) or Unicycler with default parameters (FW104-10B01 and FW107-2APBS1). The contigs were polished using the Illumina reads and Pilon (53). All strains were circularized. The longest contigs were rotated to start at either *repA* or *dnaA*. The genomes were annotated using the RAST server (54). All the genomes were deposited in NCBI GenBank (Table S2).

### Phylogenetic analysis and comparative genomic analysis.

The phylogenetic relationships were characterized by analyzing 16S rRNA gene similarity, average amino acid identity (AAI), and average nucleotide identity (ANI). Phylogenetic tree based on 16S rRNA gene sequences was constructed using the neighbor-joining method in MEGA V6.0 with 1,000 bootstrap iterations (55). Multiple genome alignment of the conserved genes was constructed using MAUVE (56). Comparative genome analysis of the annotated genomes was performed on EDGAR website (57), which generates a synteny plot, along with information on average AAI and ANI. The pan and core genome curves were generated using the Bacterial Pan-Genome Analysis Pipeline (BPGA) (58). The size of the pan-genome was estimated by a power law regression function *Ps* = κ*n*^γ^ using the BPGA pipeline, where *Ps* is the total number of nonorthologous gene families within its pan-genome, *n* is the number of the tested strains, κ and γ are free parameters (26). If γ < 0, the pan-genome is closed and its size reaches a constant with the addition of new genomes; if 0 < γ < 1, the pan-genome size grows continuously. The size of the core-genome was calculated according to an exponential decay function *Fc* = κ_c_exp(−n/τ_c_) + Ω in the BPGA pipeline, where *Fc* is the number of core gene families and κ_c_, τ_c_, and Ω are free parameters (26).

### Identification and analysis of genes involved in denitrification, heavy metal resistance, genomic islands, and restriction-modification system.

Denitrifying genes were manually identified according to previous work by Philippot (30). The tertiary structure of the key proteins involved in denitrification processes were predicted by RaptorX (59). Heavy metal resistance genes were identified against the BacMet database with experimentally confirmed metal resistance functions (60). The protein sequence identity comparisons of metal resistance genes were conducted using PATRIC 3.6.5 (61). Genomic islands (GIs) were predicted using IslandViewer4 (62). The genes in GIs were annotated according to EggNOG v5.0 database (63). Restriction-modification (RM) system genes were identified using BLASTP alignments against the REBASE database (64).

### Identification of methylation sites.

Guppy3 toolkits were used for base calling of the nanopore sequences with default setting (65). Minimap2 was used to align the nanopore DNA reads to the assembly of Illumina reads (66). MarginStats was used to calculate the alignment identities, and the custom script within marginAlign was used to calculate the substitutions (67). Detection of 5mC was conducted as described by Simpson et al. (68). Briefly, an index file was created to link the read IDs to their signal-level data in the fast5 files, then the base called reads were aligned to the reference genome assembled with Illumina reads and a sorted bam file was obtained after processing with SAMtools. Nanopolish call-methylation was used to detect 5mC methylation (69). To detect m6A methylation, mCaller was used. Neural network random forest naive Bayes logistic regression was used as the classifier in mCaller, which has improved accuracy compared to the methods based on deviations between measured and expected current (70).

### Calculation of the ratios of nonsynonymous substitutions to synonymous substitutions.

The average nonsynonymous substitutions (dN), synonymous substitutions (dS), and dN/dS were identified and calculated for all orthologs shared in all strains (coverage ≥ 70%, identity ≥ 80%). Briefly, the gene sequences from each strain were first aligned using Clustalw software (71), then dN, dS and dN/dS ratio were calculated using KaKs_Calculator 2.0 with NG method with FW107-2ABPS1 as the reference sequence (72). The gene was subjected to positive selection if dN/dS > 1, negative selection (purifying selection) if dN/dS < 1, and neutral selection if dN/dS = 1.
