# Supplementary material for: Genomic Features and Pervasive Negative Selection in Rhodanobacter Strains Isolated from Nitrate and Heavy Metal Contaminated Aquifer
Source: Microbiol Spectr. 2022 Feb 2;10(1):e02591-21. doi: 10.1128/spectrum.02591-21 (PMC8809349; doi:10.1128/spectrum.02591-21)
Supplement: SUPPLEMENTAL FILE 1 — Supplemental material. Download SPECTRUM02591-21_Supp_1_seq2.pdf, PDF file, 0.5 MB [file spectrum02591-21_supp_1_seq2.pdf]

Supplementary materials for

Genomic features and pervasive negative selection in *Rhodanobacter* strains isolated from nitrate and heavy metal contaminated aquifer

Mu Peng[a,*], Dongyu Wang[b,*], Lauren M. Lui[c], Torben Nielsen[c], Renmao Tian[a], Megan L. Kempher[a], Xuanyu Tao[a], Chongle Pan[b], Romy Chakraborty[d], Adam M. Deutschbauer[c,e], Michael P. Thorgersen[f], Michael W. W. Adams[f], Matthew W. Fields[g,h], Terry C. Hazen[i,j], Adam P. Arkin[c,k], Aifen Zhou[a,#], Jizhong Zhou[a,d, l,m#]

[a] Institute for Environmental Genomics, Department of Microbiology and Plant Biology, University of Oklahoma, Norman, OK, USA

[b] Department of Microbiology and Plant Biology, University of Oklahoma, Norman, OK, USA

[c] Environmental Genomics and Systems Biology Division, Lawrence Berkeley National Laboratory, Berkeley, California, USA

[d] Earth and Environmental Sciences Area, Lawrence Berkeley National Laboratory, Berkeley, California, USA

[e] Department of Plant and Microbial Biology, University of California, Berkeley, California, USA

[f] Department of Biochemistry and Molecular Biology, University of Georgia, Athens, Georgia, USA

[g] Department of Microbiology and Cell Biology, Montana State University, Bozeman, MT, USA.

[h] Center for Biofilm Engineering, Montana State University, Bozeman, MT, USA

[i]Department of Civil and Environmental Engineering, University of Tennessee, Knoxville, TN, USA

[j] Oak Ridge National Laboratory, Bioscience Division, Oak Ridge, TN, USA

[k] Department of Bioengineering, University of California, Berkeley, California, USA

[l] State Key Joint Laboratory of Environment Simulation and Pollution Control, School of Environment, Tsinghua University, Beijing, China

[m] School of Civil Engineering and Environmental Sciences, University of Oklahoma, Norman, OK, USA

Running Title: Genomic features underlying adaptation of *Rhodanobacter*

*These authors contributed equally to this work

#Address correspondence to Aifen Zhou, Aifen.Zhou-1@ou.edu and Jizhong Zhou, jzhou@ou.edu

LEGENDS FOR SUPPLEMENTAL MATERIAL

FIG S1 Synteny plot of *Rhodanobacter* genomes with FW107-2APBS1 as the reference genome. Each strain is represented by one color line, and different segments indicate the linear relationship of gene segments. The position in the reference genome is show on the x-axis, the relative positions in target genomes shown on the y-axis. Dots indicate the stop positions of orthologous genes.

FIG S2 COG expansion of pan-genome (orange circle) and core genome (grey circle) in *Rhodanobacter* genomes. The pairs of numbers are the number of core genes/ the numbers of pan genes.

FIG S3 Quality of the nanopore long reads. The average identity per aligned nanopore read to the Illumina assemblies is above 0.96 (A) with the read accuracy is higher than 97% (B).

FIG S4 Restriction enzyme digestion patterns of the genomic DNA or plasmid DNA isolated from *R. denitrificans* strains or *E. coli*. M: molecular marker. C: control, DNA samples without addition of restriction enzymes.

TABLE S1 *Rhodanobacter* strains and the geochemical properties of the isolation wells

TABLE S2 General genomic features of the *Rhodanobacter* genomes

TABLE S3 Sequence differences between FW107-2APBS1 in this study and 2APBS1 in NCBI database

TABLE S4 ANI and AAI identity matrix of eight *Rhodanobacter* genomes

TABLE S5 Summary of putative genes related to metal resistance in eight *Rhodanobacter* strains

TABLE S6 Summary of the nanopore sequence data

TABLE S7 Information of 5mC methylation sites in four *R. denitrificans* strains

TABLE S8 Information of 6mA methylation sites in four *R. denitrificans* strains

TABLE S9 dN/dS ratios of denitrification genes, metal resistance genes, and restriction-modification genes in eight *Rhodanobacter* strains

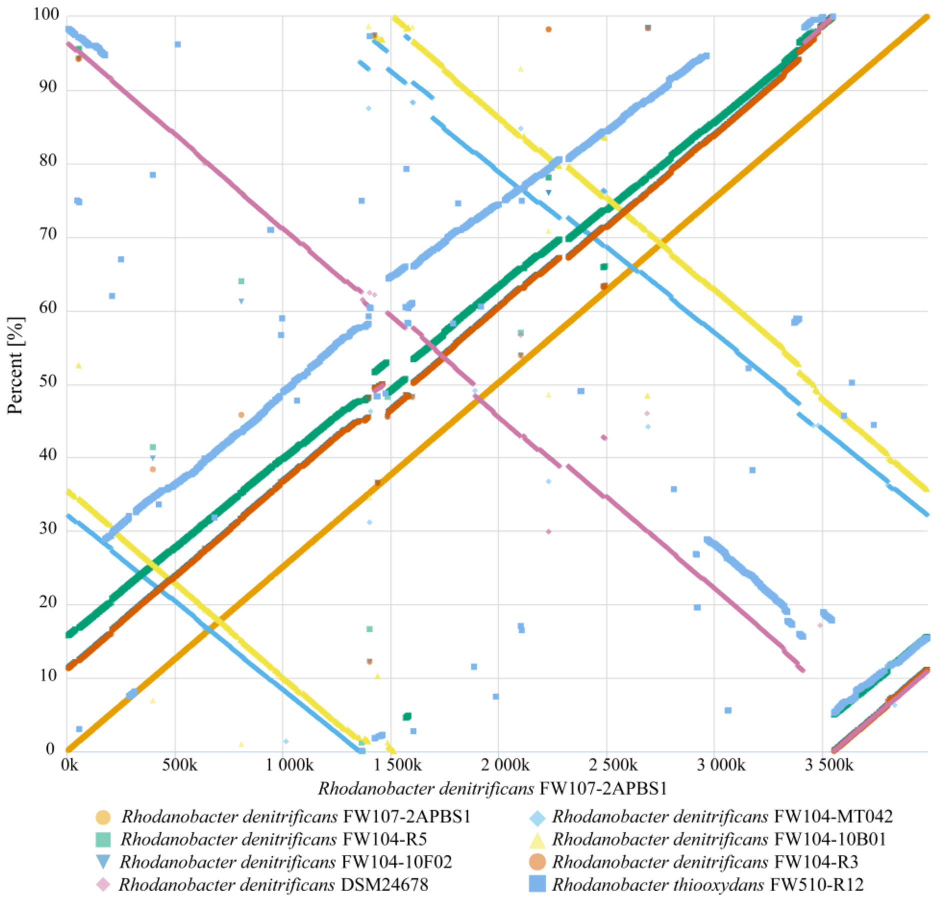

FIG S1 Synteny plot of *Rhodanobacter* genomes with FW107-2APBS1 as the reference genome. Each strain is represented by one color line, and different segments indicate the linear relationship of gene segments. The position in the reference genome is show on the x-axis, the relative positions in target genomes shown on the y-axis. Dots indicate the stop positions of orthologous genes.

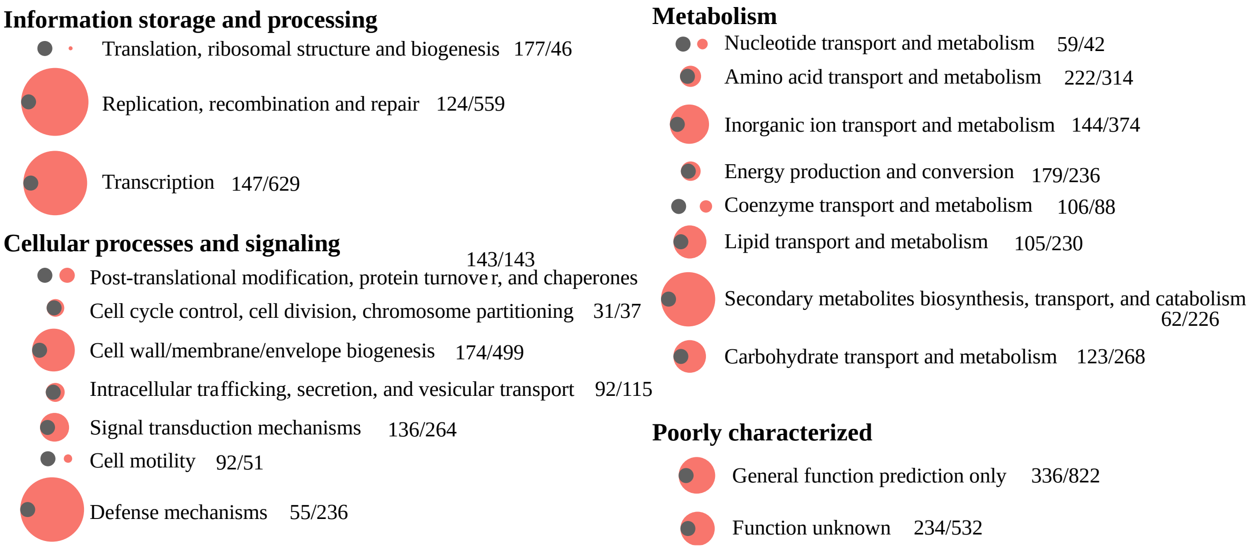

FIG S2 COG expansion of pan-genome (orange circle) and core genome (grey circle) in *Rhodanobacter* genomes. The pairs of numbers are the number of core genes/ the numbers of pan genes.

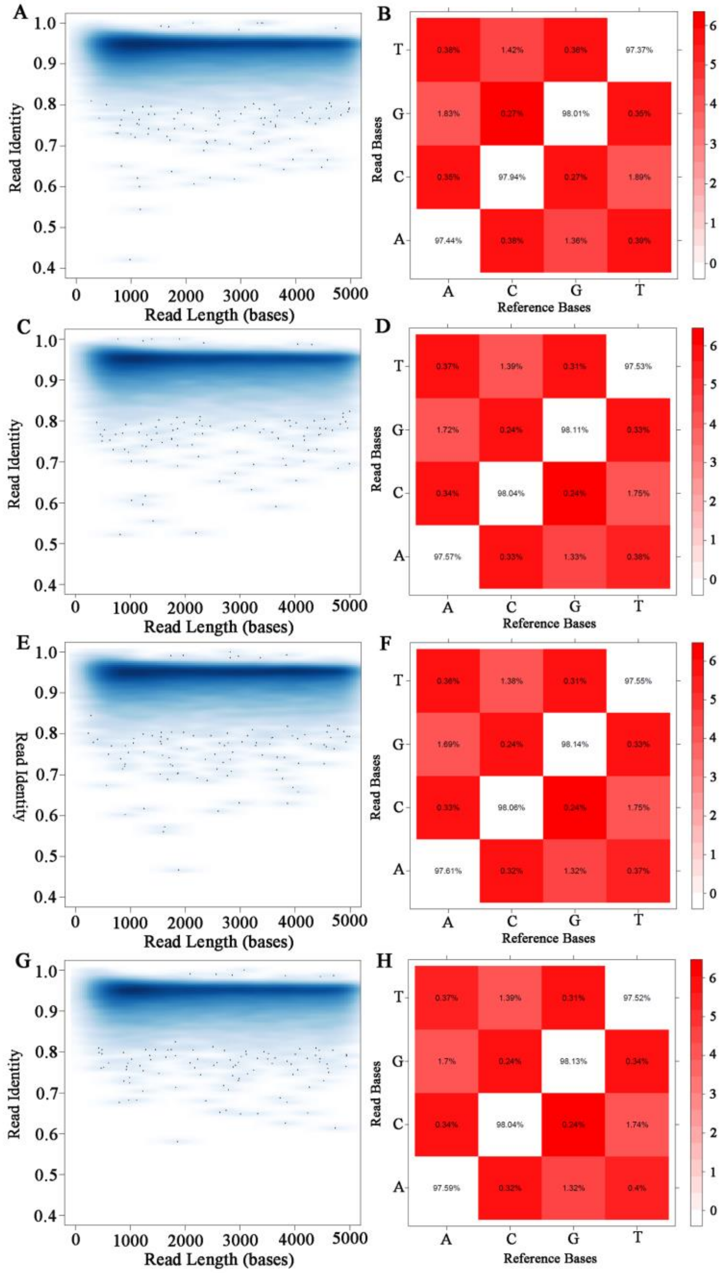

FIG S3 Quality of the nanopore long reads. The average identity per aligned nanopore read to the Illumina assemblies is above 0.96 (A) with the read accuracy is higher than 97% (B).

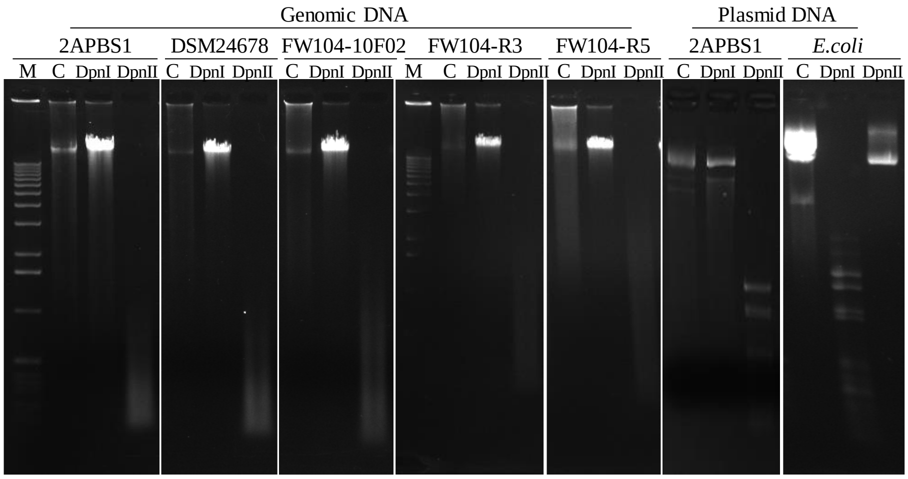

FIG S4 Restriction enzyme digestion patterns of the genomic DNA or plasmid DNA isolated from *R. denitrificans* strains or *E. coli*. M: molecular marker. C: control, DNA samples without addition of restriction enzymes.

TABLE S1 *Rhodanobacter* strains and the geochemical properties of the isolation wells

Strain name	Isolation condition	Isolation well	pH	Nitrate (mM)	Sulfate (mM)	Uranium (mM)
FW107-2APBS1*	Minimal synthetic ground water medium, anaerobic	FW107, moderately contaminated area 2	6~7	<2	N/A	N/A
DSM-24678#	Potato dextrose	Most highly contaminated area 3	3~4	10~100	N/A	N/A
FW104-10B01	R2A, aerobic, 30°C	FW104	5~6	90~150	3~60	0.04~0.14
FW104-MT042	R2A, aerobic, 30°C	FW104	5~6	90~150	3~60	0.04~0.14
FW104-10F02	R2A, aerobic, 30°C	FW104	5~6	90~150	3~60	0.04~0.14
FW104-R3	R2A, aerobic, 30°C	FW104	5~6	90~150	3~60	0.04~0.14
FW104-R5	R2A, aerobic, 30°C	FW104	5~6	90~150	3~60	0.04~0.14
FW510-R12	R2A+Mn, aerobic, 30°C	FW510	4	14	<1	0.004

*: complete genome sequence is available in NCBI before this work, type strain. #: purchased from DSMZ

TABLE S2 General genomic features of the *Rhodanobacter* genomes

	DSM24678	FW104-10F02	FW104-R3	FW104-R5	FW104-10B01	FW510-R12	FW104-MT042	FW107-2APBS1
Size (base pairs)	3,958,367	3,959,114	3,959,116	4,134,749	3,959,115	4,203,948	4,236,788	3,984,560
GC content (%)	67.66	67.65	67.65	67.62	67.65	67.09	67.36	67.58
Protein-coding genes	3,707	3,695	3,704	3,909	3,706	3,928	4,035	3,786
Proteins with functional assignments	2,672	2,676	2,676	2,752	2676	2,724	2,762	2,712
Proteins with Enzyme Commission number assignments	896	895	895	905	895	923	920	903
Proteins with Gene Ontology assignments	760	755	755	759	755	778	779	764
Proteins with Pathway assignments	652	650	650	654	650	689	665	650
Antibiotic Resistance genes	36	36	36	36	36	36	38	35
Virulence factor genes	1	1	1	1	1	1	1	1
tRNA	49	49	49	49	49	49	50	49
rRNA	6	6	6	6	6	6	6	6
No. of plasmids	0	0	0	1	0	1	1	0
Sequencing platform	Illumina and nanopore	Illumina and nanopore	Illumina and nanopore	Illumina and nanopore	Illumina and pacbio	Illumina and pacbio	Illumina and pacbio	Illumina and pacbio
NCBI accession number	CP088919	CP088920	CP088921	CP088980 & CP088981	CP088922	CP088923 & CP088924	CP088925 & CP088926	CP088918

TABLE S3 Sequence differences between FW107-2APBS1 in this study and 2APBS1 in NCBI database

Type of the difference	Location of the difference	Position of the difference	Nucleotide change	Amino acid change	Affected gene(s)
SNP	Intergenic	2,659	T→C	No	No
SNP	Gene	398,426	T→G	No	Hypothetical protein
Del	Intergenic	420,688	-2: GC	No	No
SNP	Gene	528,218	T→A	Ter→Lys	Signal transduction histidine kinase CheA
Del	Intergenic	990,568	-706	No	No
Ins	Intergenic	991,273	+485	No	No
SNP	Gene	1,090,662	G→C	No	Hypothetical protein
Del	Intergenic	1,337,608	-1: T	No	No
Del	Intergenic	1,337,750	-1: T	No	No
Del	Gene	1,472,181	-1: T	380 more amino acids	CzcABC family efflux RND transporter, transmembrane protein
Del	Gene	1,578,104	-241,020	-71,201 amino acids	256 genes missing
SNP	Gene	1,989,582	T→C	Thr→Ala	Mercuric reductase
SNP	Gene	2,268,696	C→A	Thr→Lys	ABC-type efflux pump membrane fusion component YbhG
Ins	Gene	2,346,606	+1: G	54 more amino acids	Nitrate/nitrite transporter NarK/U 1
Del	Intergenic	2,383,369	-1: A	No	No
Del	Intergenic	2,410,869	-1: T	No	No
Del	Intergenic	2,554,159	-1: A	No	No
SNP	Gene	2,781,335	A→G	Thr→Ala	LppC putative lipoprotein
SNP	Intergenic	2,908,993	T→G	No	No
SNP	Intergenic	2,909,015	T→G	No	No
Del	Intergenic	3,427,464	-1: T	No	No

TABLE S4 ANI and AAI identity matrix of eight *Rhodanobacter* genomes

	DSM24678	FW104-R5	FW104-10B01	FW104-10F02	FW104-R3	FW107-2APBS1	MT042	FW510-R12
DSM24678		98.26	98.27	98.27	98.33	98.17	98.01	88.84
FW104-R5	98.9		99.66	99.66	99.68	98.42	98.55	88.92
FW104-10B01	98.83	99.82		100	100	98.19	98.31	89.23
FW104-10F02	98.83	99.82	100		100	98.19	98.31	89.23
FW104-R3	98.83	99.82	100	100		98.19	98.31	89.23
2APBS1	98.66	98.81	98.71	98.7	98.7		98.3	88.9
FW104-MT042	98.74	98.98	98.92	98.92	98.91	98.8		88.5
FW510-R12	89.83	89.84	89.91	89.91	89.9	89.81	89.84	

Lower triangle: average amino acid identity (AAI); upper triangle: average nucleotide identity (ANI).

TABLE S6 Summary of the nanopore sequence data

Strain	Nanopore reads	Mapped nanopore reads	Mapping rate of nanopore reads	Genome size (bp)	Bases (bp) covered by nanopore reads	Genome coverage rate
DSM24678	112,602	112,427	99.84%	3,958,367	3,958,367	100.00%
FW104-10F02	72,206	71,958	99.66%	3,959,114	3,959,114	100.00%
FW104-R3	87,045	86,876	99.81%	3,959,116	3,959,116	100.00%
FW104-R5	85,530	85,234	99.65%	4,134,749	4,134,749	100.00%
